# Comparative Analysis of Diagnostic Performance Between Elastography and AI-Based S-Detect for Thyroid Nodule Detection

**DOI:** 10.3390/diagnostics15172191

**Published:** 2025-08-29

**Authors:** Jee-Yeun Park, Sung-Hee Yang

**Affiliations:** 1 Department of Radiological Science, Jangpalpal Internal Medicine Clinic, 369, Haeundae-ro, Haeundae-gu, Busan 48062, Republic of Korea; roks73@naver.com; 2 Department of Radiological Science, College of Health Sciences, Catholic University of Pusan, Busan 46252, Republic of Korea

**Keywords:** thyroid nodule, benign, malignant, elastography, thyroid ultrasonography

## Abstract

**Background/Objectives:** Elastography is a non-invasive imaging technique that assesses tissue stiffness and elasticity. This study aimed to evaluate the diagnostic performance and clinical utility of elastography and S-detect in distinguishing benign from malignant thyroid nodules. S-detect (RS85) is a deep learning-based computer-aided diagnosis (DL-CAD) software that analyzes grayscale ultrasound 2D images to evaluate the morphological characteristics of thyroid nodules, providing a visual guide to the likelihood of malignancy. **Method:** This retrospective study included 159 patients (61 male and 98 female) aged 30–83 years (56.14 ± 11.35) who underwent thyroid ultrasonography between January 2023 and June 2024. All the patients underwent elastography, S-detect analysis, and fine needle aspiration cytology (FNAC). Malignancy status was determined based on the FNAC findings, and the diagnostic performance of the elasticity contrast index (ECI), S-detect, and evaluations by a radiologist were assessed. Based on the FNAC results, 101 patients (63.5%) had benign nodules and 58 patients (36.5%) had malignant nodules. **Results:** Radiologist interpretation demonstrated the highest diagnostic accuracy (area under the curve 89%), with a sensitivity of 98.28%, specificity of 79.21%, positive predictive value (PPV) of 73.1%, and negative predictive value (NPV) of 98.8%. The elasticity contrast index showed an accuracy of 85%, sensitivity of 87.93%, specificity of 81.19%, PPV of 72.9%, and NPV of 92.1%. S-detect yielded the lowest accuracy at 78%, with a sensitivity of 87.93%, specificity of 68.32%, PPV of 61.4%, and NPV of 90.8%. **Conclusions:** These findings offer valuable insights into the comparative diagnostic utility of elastography and AI-based S-detect for thyroid nodules in clinical practice. Although limited by its single-center design and sample size, which potentially limits the generalization of the results, the controlled environment ensured consistency and minimized confounding variables.

## 1. Introduction

Thyroid cancer is one of the most common endocrine malignancies worldwide. According to the International Agency for Research on Cancer (IARC) under the World Health Organization (WHO), thyroid cancer accounts for approximately 87,000 new cases annually worldwide, with an incidence rate ranging from 0.5 to 10 cases per 100,000 people. With the increasing prevalence of high-resolution ultrasound and routine medical examinations, the detection rate of thyroid nodules has significantly risen. Thyroid nodules are common clinical findings characterized by the overgrowth of thyroid cells and are detected in approximately 19–67% of the population through thyroid ultrasound. Approximately 5–15% of these cases are diagnosed as thyroid cancer [1,2,3]. As the detection of thyroid nodules increases, the frequency of thyroid cancer is also steadily increasing. Although the occurrence of thyroid nodules cannot be completely prevented, regular health check-ups enable early detection and management.

The standard diagnostic approach for thyroid nodules includes physical examination, laboratory assessments, thyroid nuclear scans, imaging modalities such as ultrasonography, and confirmatory procedures such as fine needle aspiration cytology (FNAC). Additional imaging techniques, such as computed tomography and magnetic resonance imaging, may be used if necessary [3,4,5,6]. Ultrasonography, in particular, is a highly useful, non-invasive, and convenient method for diagnosing thyroid lesions, as it not only detects nodules but also evaluates the involvement of surrounding lymph nodes and adjacent tissues [7]. The main disadvantage of the method is that it is operator-dependent. Despite its high sensitivity in assessing malignancy risk, ultrasonography has limited specificity, and further evaluation using FNAC is required to confirm malignancy [1,8].

To standardize the interpretation of thyroid ultrasound findings and improve diagnostic consistency, several radiological assessment systems have been developed. Among these, the Thyroid Imaging Reporting and Data System (TIRADS) is widely used. TIRADS classifies nodules based on specific sonographic features (such as composition, echogenicity, margins, shape, and calcifications) and assigns a malignancy risk score to guide clinical decision-making. These systems aim to reduce inter-observer variability and support appropriate recommendations for FNAC or follow-up [9].

Although ultrasound is an excellent test for the diagnosis of thyroid nodules, there is a growing enthusiasm for innovative diagnostic techniques that can enhance both sensitivity and specificity. There is a high interest in a better way to detect cancer with precision while minimizing unnecessary testing, thereby refining patient care. Performing FNAC on all nodules detected by ultrasonography is impractical, as it may increase healthcare costs due to unnecessary testing and cause patient discomfort from invasive procedures. Although FNAC is useful for distinguishing between benign and malignant thyroid nodules, performing it on all nodules is considered an unnecessary utilization of healthcare resources [10,11]. FNAC is a safe, simple, and cost-effective diagnostic method for evaluating thyroid nodules, playing a critical role in the selection of surgical, interventional, or conservative management [12]. However, there has been a problem with over-diagnosis and over-treatment due to the slow growth and lower invasiveness of many thyroid cancers [13].

To enhance diagnostic accuracy and reduce unnecessary interventions, advanced imaging methods such as elastography and AI-based diagnostic tools have been introduced [14]. Elastography is a real-time, non-invasive imaging technique that assesses tissue stiffness by measuring the degree of deformation under applied pressure. This advanced computer-aided diagnosis (CAD) technology operates on the principle that softer areas deform more easily than firmer areas when pressure is applied [15]. The ECI quantitatively assesses the stiffness contrast between the nodule and surrounding normal tissue and is a useful index for the evaluation of malignancy risk [16].

Meanwhile, S-detect is a deep learning-based computer-aided diagnosis (DL-CAD) system that analyzes grayscale 2D ultrasound images to evaluate the morphological characteristics of thyroid nodules, providing a visual guide to the likelihood of malignancy [17,18]. Both ECI and S-detect contribute to a more precise characterization of thyroid nodules and facilitate appropriate treatment planning by providing data on the elasticity and morphological characteristics of nodules, respectively. These technologies offer objective, reproducible data that may overcome the limitations of subjective visual interpretation in conventional ultrasound.

Ultrasonography of the thyroid relies on visual assessment, meaning that the accuracy of diagnosis can vary based on the experience and skill of the examiner. We hypothesize that both ECI and S-detect have the potential to serve as adjunctive or alternative diagnostic tools to FNAC in selected clinical scenarios, especially where non-invasive decision-making is prioritized. This study aims to evaluate the diagnostic performance and clinical utility of these modalities in differentiating between benign and malignant thyroid nodules. Furthermore, we seek to explore how their implementation could contribute to reducing the number of unnecessary invasive procedures and ultimately improve diagnostic efficiency and patient care.

## 2. Materials and Methods

### 2.1. Study Design and Population

This retrospective, single-center study was conducted at J Internal Medicine Clinic in Busan, Republic of Korea, between January 2023 and June 2024. A total of 194 patients with newly diagnosed thyroid nodules underwent initial evaluations, including thyroid ultrasonography and fine needle aspiration cytology (FNAC).

Inclusion criteria were as follows:

- Adults aged 30–83 years;
- Newly diagnosed with one or more thyroid nodules;
- Underwent FNAC with conclusive cytological results;
- (Bethesda categories II, IV, V, or VI).

Exclusion criteria were as follows:

- Prior history of thyroid surgery;
- Ongoing treatment for thyroid-related conditions;
- FNAC results categorized as Bethesda I;
- (Non-diagnostic due to insufficient specimen);
- FNAC results categorized as Bethesda III;
- (Atypia/follicular lesion of undetermined significance).

**Figure 1:**
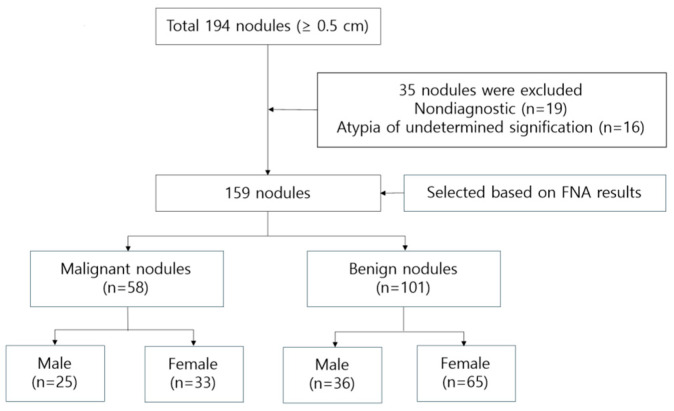
Flowchart of the study population.

After applying these criteria, 159 patients (61 males and 98 females) were included in the final analysis. The study’s flowchart is depicted in Figure 1.

The participants underwent FNAC after a new diagnosis of one or more thyroid nodules. Patients who had previously undergone thyroid surgery, were currently undergoing treatment for thyroid-related conditions, or had nondiagnostic or unsatisfactory FNAC results due to inadequate sampling were excluded from the study [19]. The FNAC results were interpreted according to ‘The Bethesda System for Reporting Thyroid Cytopathology’, which classifies malignancy risk into six categories [20]. Each category has an implied cancer risk, which ranges from 1% to 3% overall for the “Benign” category to virtually 100% for the “Malignant” category [21]. Each category, based on its associated cancer risk, is linked to evidence-based clinical management guidelines, as outlined in Table 1.

Ethical approval for this study was obtained from the Institutional Bioethics Committee of University B on 24 June 2024 (approval NO. CUPIRB-2024-033).

### 2.2. Ultrasound Examination

**Figure 2:**
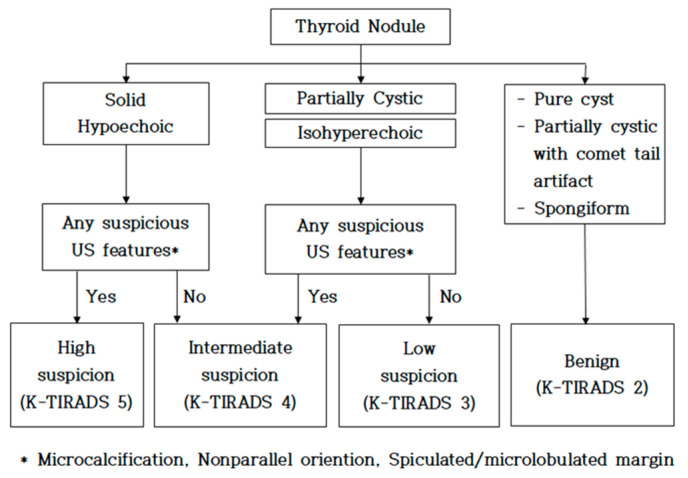
An algorithm of K-TIRADS for malignancy risk stratification based on solidity and echogenicity of thyroid nodules.

Thyroid nodule detection was performed using a high-resolution ultrasound system (RS85, Samsung Medison, Seoul, Republic of Korea) with a 14 MHz high-frequency linear transducer (LA 2-14A, Samsung Medison). An ultrasound technologist with over 20 years of clinical experience conducted the ultrasound examinations, and a radiologist interpreted the images based on the guidelines of the Korean Thyroid Imaging Reporting and Data System (K-TIRADS). K-TIRADS is a standardized ultrasound-based risk stratification system developed by the Korean Society of Thyroid Radiology. It categorizes thyroid nodules into five groups based on sonographic features such as echogenicity, composition, shape, margin, and the presence of calcifications. Sonographic findings were classified based on the echogenicity and morphological characteristics of the thyroid nodules. Malignant features included irregular or lobulated margins, microcalcifications, and a taller-than-wide shape in the transverse view. Nodules corresponding to K-TIRADS categories 2 and 3 were classified as benign, whereas those falling into categories 4 and 5 were considered malignant. An internal medicine specialist with 20 years of experience performed the FNAC procedure according to the K-TIRADS guidelines. Each case involved more than three punctures, and the final diagnoses were confirmed by a certified pathologist [22,23]. The K-TIRADS algorithm for malignancy risk is shown in Figure 2.

### 2.3. Elastography

**Figure 3:**
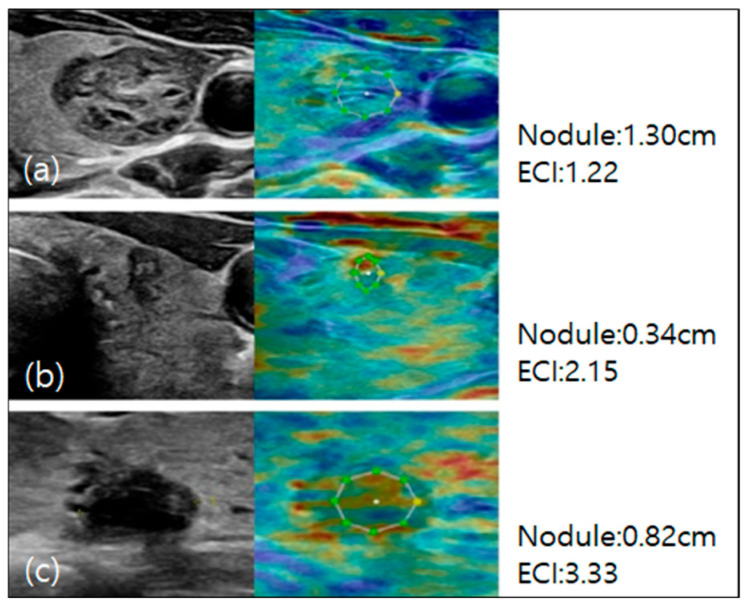
ECI image of thyroid nodule. The color map reflicts elasticity (blue: low stiffness, green: intermediate, red: high stiffness). The dotted circle marks the lesion boundary, and the solid circle indicates the ROI used for ECI ratio calculation. (**a**) Isoechoic, well-defined margin and spongiform nodule localized in the left lobe of the thyroid gland. The axial peri-intranodular ECI was 1.22. Histology confirmed the diagnosis of benign nodular hyperplasia. (**b**) Hypoechoic nodule with taller than wider and some internal calcifications localized in the left lobe of the thyroid gland. The axial peri-intranodular ECI was 2.15. Histology confirmed the diagnosis of papillary carcinoma. (**c**) Hypoechoic nodule with irregular margins and some internal calcifications localized in the middle portion of the thyroid gland. Peri-intranodular ECI measures 3.33. Histology confirmed the diagnosis of papillary carcinoma.

**Figure 4:**
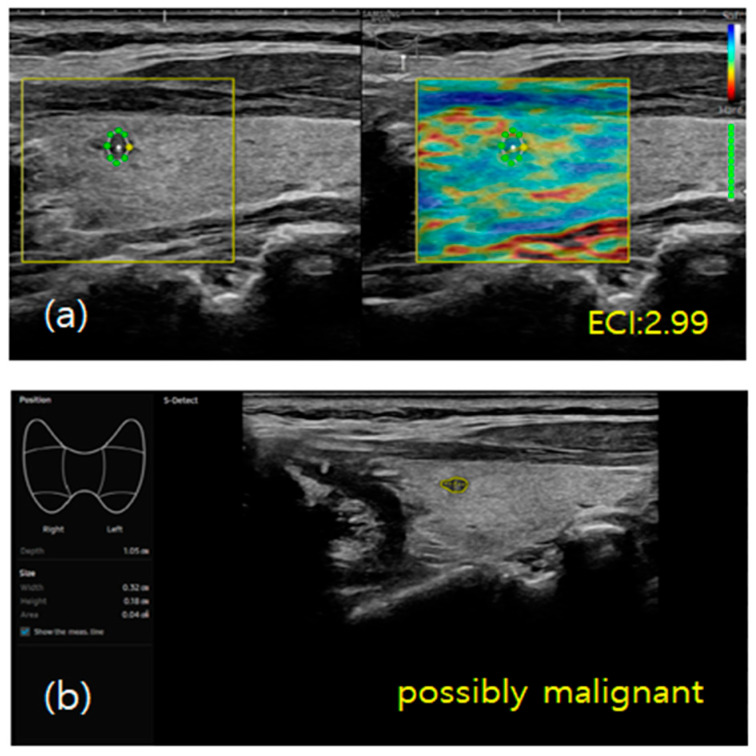
Hypoechoic nodule with irregular margins localized in the upper portion of the thyroid gland. (**a**) Peri-intranodular ECI measures 2.99. (**b**) S-detect indicated “possibly malignant”.

For the elastography procedure, the patients were positioned supine with their heads slightly turned away from the thyroid being examined and their chins extended. B-mode ultrasound images of the thyroid nodule were acquired, and static elastography was performed [24]. Images were obtained by using light pressure to ensure patient comfort, and ECI was automatically measured within the region of interest. The ECI is derived using the E-Thyroid software (RS85, Samsung Medison), which employs a steady-state quasi-static physiological excitation technique. This method leverages the carotid pulsation as a strain inducer to achieve a quantitative evaluation of tissue stiffness [25]. The relevant case is depicted in Figure 3 and Figure 4.

### 2.4. S-Detect

**Figure 5:**
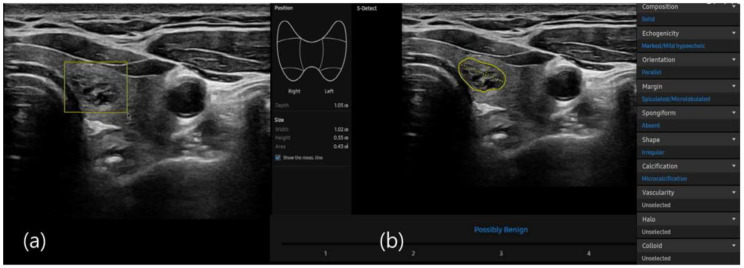
(**a**) Ultrasound grayscale transverse image of the left thyroid lobe in a 57-year-old woman. (**b**) Thyroid nodules automatically classified as “possibly benign” using S-detect according to characteristics such as internal composition, echogenicity, orientation, margin, and shape.

**Figure 6:**
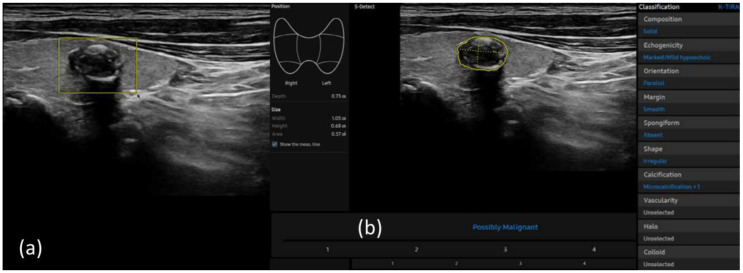
(**a**) Ultrasound grayscale longitudinal image of the left thyroid lobe in a 69-year-old man. (**b**) Thyroid nodules automatically classified as “possible malignant” using S-detect according to characteristics such as internal composition, echogenicity, orientation, margin, and shape.

S-detect is an AI-based CAD software designed for ultrasound image analysis. Upon establishing the region of interest (ROI) for the targeted nodule, the software automatically delineates the boundaries and initiates the analysis [22,26]. If there were errors in the boundaries delineated by the software, manual delineation of the boundaries was performed. First of all, conventional ultrasound scanning was performed. After freezing a static ultrasound image of the thyroid nodule, the ROI was manually set around the lesion, and S-detect was subsequently utilized to analyze the lesions. The nodules were then classified as “possibly benign” or “possibly malignant” based on characteristics such as internal composition, echogenicity, orientation, margin, and shape. All classifications rendered by S-detect (“possibly benign” or “possibly malignant”) were independently reviewed by an internal medicine specialist with over 20 years of experience in thyroid imaging. This procedure is depicted in Figure 5 and Figure 6.

We also noted that the S-detect software (Samsung Medison Co., Ltd.) used in this study has been validated in previous studies that demonstrated its diagnostic reliability and accuracy in differentiating benign from malignant thyroid nodules [17,22]. These prior studies served as the basis for its clinical implementation in our study setting.

### 2.5. Statistical Analysis

The general characteristics of the study population are presented as mean and standard deviation (SD) or number of persons (*n*) and percentage (%). The necessity of the ROC curve lies in finding a classification model that achieves a suitable balance while minimizing false positives (predicting a negative as positive) and false negatives (predicting a positive as negative). To assess the diagnostic utility of elastography, receiver operating characteristic (ROC) curve analysis was conducted, and the area under the curve (AUC), positive predictive value (PPV), negative predictive value (NPV), sensitivity, and specificity were calculated. For reliability assessment, diagnostic agreement with FNAC results was evaluated using Cohen’s Kappa analysis. Statistical significance was set at *p* < 0.05, and all analyses were performed using SPSS Version 29.0 (IBM Corp., Armonk, NY, USA).

## 3. Results

### 3.1. General Characteristics of Patients

Based on the FNAC results, 101 patients (63.5%) had benign nodules, and 58 patients (36.5%) had malignant nodules. The cohort included 61 (38.4%) males and 98 (61.6%) females, with no significant difference in sex distribution between the benign and malignant groups (*p* = 0.352). The mean age of the study population was 56.14 ± 11.35 years. The nodules’ average size was confirmed to be 1.07 ± 0.85 cm, with a statistically significant difference observed between benign (1.23 ± 0.94 cm) and malignant (0.79 ± 0.58 cm) nodules (*p* = 0.002). In terms of nodule composition, 146 (91.8%) were solid, 11 (6.9%) were predominantly solid, and 2 (1.3%) were predominantly cystic, with no significant difference between the benign and malignant groups (*p* = 0.077). Regarding echogenicity, 119 nodules (74.8%) were hypoechoic, and 40 (25.2%) were isoechoic; no hyperechoic nodules were identified (*p* < 0.001). Regarding nodule orientation, 105 (66.0%) were parallel, and 54 (34.0%) were nonparallel, with nonparallel orientation being more frequently observed in malignant cases (*p* < 0.001). In terms of nodule margins, 75 nodules (47.2%) were circumscribed, and 84 (52.8%) were not circumscribed, with circumscribed margins being more common in benign nodules and not circumscribed margins in malignant ones (*p* < 0.001). Regarding shape, 67 nodules (42.1%) were oval, 34 (21.4%) were round, and 58 (36.5%) were irregular, with irregular shapes being more frequently associated with malignancy (*p* < 0.001). Calcification was present in 111 nodules (69.8%) and absent in 48 (30.2%), showing a significant difference between the two groups (*p* < 0.001). Posterior shadowing was observed in 131 nodules (82.4%), while 28 (17.6%) showed no shadowing (*p* = 0.021). According to the S-detect analysis, 76 nodules (47.8%) were classified as having benign nodules, while 83 nodules (52.2%) were classified as malignant. The ECI results identified 89 nodules (56.0%) as benign and 70 nodules (44.0%) as malignant. The radiologist’s findings, considering the K-TIRADS characteristics, showed 81 nodules (50.9%) with benign nodules and 78 nodules (49.1%) with malignant nodules. The results are detailed in Table 2.

### 3.2. Performance of Diagnostic Models

**Figure 7:**
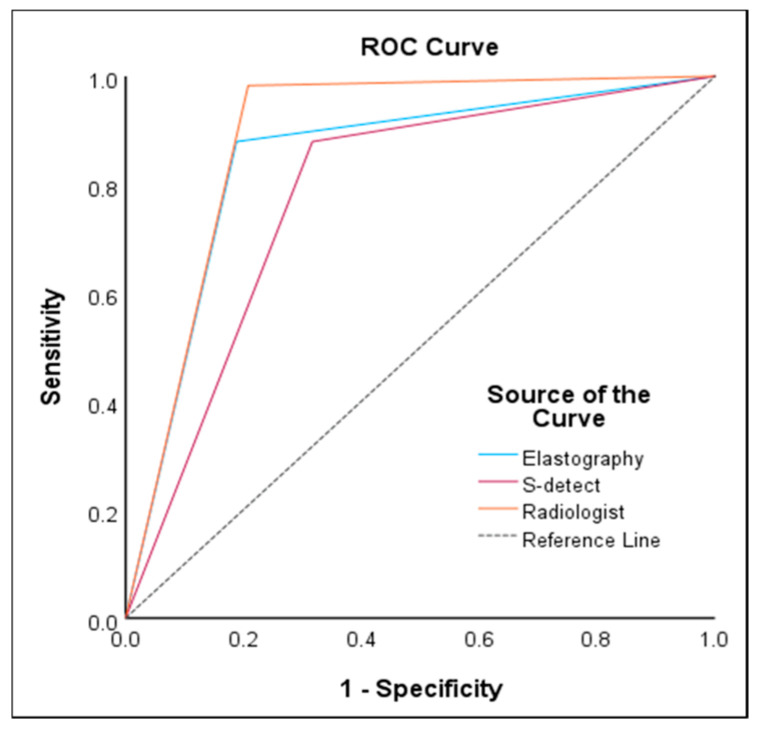
Receiver operating characteristic curve analysis comparing the diagnostic performance of the radiologist, S-detect, and elastography based on fine needle aspiration cytology results.

In the ROC curve analysis, S-detect had an AUC of 0.78 (95% CI 0.72–0.84, *p* < 0.001), sensitivity of 87.93%, specificity of 68.32%, PPV of 61.4%, and NPV of 90.8%. For ECI, the optimal cutoff value was 2.41 and had an AUC of 0.85 (95% CI 0.79–0.90, *p* < 0.001), sensitivity of 87.93%, specificity of 81.19%, PPV of 72.9%, and NPV of 92.1%. Radiologist evaluation had an AUC of 0.89 (95% CI 0.84–0.93, *p* < 0.001), sensitivity of 98.28%, specificity of 79.21%, PPV of 73.1%, and NPV of 98.8%. The results are presented in Table 3 and Figure 7.

### 3.3. Agreement of Diagnostic Models

To evaluate the diagnostic agreement of each modality with the reference standard (FNAC), Cohen’s Kappa coefficients were calculated. Based on the widely accepted classification by Landis and Koch, Kappa values were interpreted as follows: values of 0.41–0.60 indicate moderate agreement, and 0.61–0.80 indicate substantial agreement. According to this scale, S-detect exhibited moderate agreement (Kappa value of 0.52, 95% CI 0.38–0.64, *p* < 0.001), while ECI showed substantial agreement (Kappa value of 0.66, 95% CI 0.54–0.78, *p* < 0.001). K-TIRADS evaluation by an internist also demonstrated substantial agreement (Kappa value of 0.72, 95% CI 0.62–0.83, *p* < 0.001) [27]. These findings suggest that both ECI and K-TIRADS achieved higher diagnostic consistency with FNAC compared to S-detect. All agreements were statistically significant, underscoring the clinical reliability of these modalities. The detailed results are summarized in Table 4.

## 4. Discussion

In this study, we demonstrated that elastography exhibited superior diagnostic performance compared to the AI-based S-detect system in differentiating benign and malignant thyroid nodules. Specifically, the ECI showed higher diagnostic concordance (0.66) than S-detect (0.52), suggesting that elastography may serve as a more reliable adjunct tool in clinical practice.

These findings are consistent with previous studies indicating that elastography, by quantifying tissue stiffness, enhances the sensitivity and specificity of thyroid nodule evaluation [22]. Moreover, recent research integrating shear wave elastography with computer-aided diagnosis systems has shown improved accuracy in malignancy detection, reducing both missed diagnoses and unnecessary biopsies [16].

Thyroid nodules are generally slow-growing and often do not require surgical intervention. According to the National Cancer Registry Statistics in the Republic of Korea, the 5-year relative survival rate (2016–2020) for thyroid cancer is 100% [28]. With early detection and appropriate treatment, malignant thyroid nodules have a favorable prognosis compared to other cancers [29]. Additionally, avoiding unnecessary tests and surgeries for benign nodules helps reduce healthcare costs and improves patients’ quality of life [30,31]. Therefore, distinguishing between benign and malignant nodules at an early stage is crucial.

In recent years, artificial intelligence AI-based computer-aided diagnostic (CAD) systems have been introduced to reduce interobserver variability and improve diagnostic consistency in thyroid ultrasound examinations [32,33]. In this study, we compared the diagnostic performance of radiologist evaluation (K-TIRADS) with AI-based S-detect and ECI (elastography) to examine their potential as adjunctive diagnostic tools.

In this study, the diagnostic accuracy was the highest for K-TIRADS-based radiologist evaluation, with an accuracy of 89%, sensitivity of 98.28%, specificity of 79.21%, PPV of 73.1%, and NPV of 98.8%. Next, the accuracy of ECI was relatively high at 85%, with a sensitivity of 87.93%, specificity of 81.19%, PPV of 72.9%, and NPV of 92.1%, indicating that ECI can be helpful in differentiating thyroid nodules. The accuracy of S-detect was the lowest among the diagnostic models, at 78%, with a sensitivity of 87.93%, specificity of 68.32%, PPV of 61.4%, and NPV of 90.8%. According to Cho et al. [25], the diagnostic accuracy of combined grayscale ultrasound and ECI was 78.6% when using an ECI cutoff value of 3.5, which was higher than that of grayscale ultrasound (76.9%) and ECI alone (67.1%). Sheng et al. [34] reported that ECI significantly increases in patients with Hashimoto’s thyroiditis and can be used to assess the degree of immune dysfunction. Di et al. [35] found that the optimal ECI cutoff value was 2.16, lower than the 2.41 found in this study, with a sensitivity of 90.3%, specificity of 82.9%, PPV of 83.7%, and NPV of 91.2%. The said study suggested that these variations could be attributed to differences in study populations or diagnostic criteria, confirming that ECI can be a useful tool for assessing the malignant potential of thyroid nodules in specific contexts. In addition, the authors of the said study suggest that elastography, which measures tissue stiffness upon external pressure in real-time through non-invasive methods, is a useful tool for differentiating thyroid nodules [35,36]. Zhou et al. [37] demonstrated that combining AI-based analysis with elastographic imaging and TIRADS classification significantly improved diagnostic performance, particularly in nodules with indeterminate sonographic features. Similarly, Zimen et al. [38] reported that integrating shear wave elastography with CAD systems reduced missed malignancy rates and unnecessary biopsies, achieving an AUC of 77.4% for nodules measuring 2–4 cm.

S-detect showed lower diagnostic accuracy compared to ECI and radiologist interpretation. This may be due to its exclusive dependence on grayscale ultrasound images, potentially limiting its ability to account for tissue stiffness or vascularity. Additionally, variations in image acquisition or nodule characteristics may affect performance. These findings are in line with previous research suggesting that AI-based tools show improved diagnostic value when used in conjunction with other imaging modalities or expert review [39,40].

While S-detect offers the advantage of automated morphological analysis based on grayscale ultrasound images, its lower specificity (68.32%) compared to elastography (81.19%) may limit its standalone utility in clinical decision-making. The discrepancy may stem from the inherent limitations of AI algorithms in interpreting subtle echogenic features and calcification patterns, which are better captured through elasticity-based imaging modalities.

A limitation of this study is the lack of an objective criterion for accurately measuring the degree of fine pressure applied during elastography, which might have led to variability in the elasticity grades assigned by each examiner [41]. In addition, the sample size was limited to 159 subjects, and the study was conducted in a single geographic area, which may limit the generalizability of the results. To address these limitations, larger-scale studies involving diverse population groups are necessary. Moreover, educational programs to enhance examiner proficiency, research aimed at improving diagnostic accuracy through the development of objective measurement criteria for elastography, and advancements in artificial intelligence-based diagnostic tools should continue. Despite these limitations, elastography has proven to be a valuable non-invasive diagnostic tool for thyroid nodules. The Kappa coefficient of 0.66 observed in this study indicates substantial agreement with FNAC results, suggesting that elastography could play an important role in the development and refinement of diagnostic techniques for thyroid nodules [42,43]. The results of this study show that ECI, as calculated through elastography, demonstrated relatively high sensitivity and specificity. Although S-detect’s accuracy was lower compared to the other two methods, it still shows promise as an artificial intelligence-based diagnostic tool for thyroid nodule evaluation.

Elastography and S-detect demonstrated superior diagnostic performance over conventional B-mode ultrasonography by enhancing tissue stiffness quantification and providing algorithm-based interpretation, respectively. These modalities may contribute to improved diagnostic accuracy and reproducibility in the evaluation of thyroid nodules, particularly those with indeterminate sonographic features.

Taken together, our comparative analysis underscores the potential of elastography as a complementary diagnostic tool, particularly in cases where conventional ultrasound and AI-based assessments yield ambiguous results. Future multi-center studies with larger cohorts are warranted to validate these findings and explore hybrid diagnostic models that integrate elastography and AI technologies.

## 5. Conclusions

This study evaluated the diagnostic performance of elastography and AI-based S-detect as adjunctive tools for thyroid nodule assessment. These technologies enable rapid, objective analysis of ultrasound images, thereby enhancing diagnostic efficiency and supporting clinicians in making more accurate and consistent decisions.

Although elastography and S-detect cannot fully replace FNAC, their combined use significantly improves the accuracy and reliability of thyroid nodule diagnosis. This may contribute to reducing unnecessary FNAC and biopsy procedures, particularly in low-risk cases, ultimately improving patient comfort and resource utilization.

However, before these tools can be widely adopted in routine clinical practice, several limitations must be addressed. These include the retrospective, single-center design of the study, operator dependency in elastography, and the limited interpretation of AI algorithms. Furthermore, the diagnostic performance in indeterminate nodules and long-term clinical outcomes was not evaluated.

To establish broader applicability, future research should include large-scale, multi-center prospective studies across diverse populations and nodule types. Technological refinements such as improved deep learning algorithms, multi-modal integration, and real-time feedback may further enhance diagnostic precision. Additionally, structured education and training programs are essential to ensure consistent and effective clinical implementation.

In conclusion, the integration of elastography and S-detect into ultrasound workflows holds promise for improving diagnostic accuracy, reducing unnecessary invasive procedures, and optimizing thyroid cancer management.

## Figures and Tables

**Table 1:** The Bethesda system for reporting thyroid cystopathology.

Category	Meaning	Risk of Malignancy (%)
I	Non-diagnostic or inadequate	1–4
II	Benign	1–3
III	Atypia/follicular lesion of undetermined significance	5–15
IV	Follicular neoplasm or suspicious for follicular neoplasm	20–30
V	Suspicious for malignancy	60–75
VI	Malignant	97–99

**Table 2:** Comparison of characteristics between benign and malignant nodules (*n* = 159).

Variable		*n* (%)	Benign	Malignant	χ^2^/t	*p* Value
Sex	Male	61 (38.4)	36 (22.7)	25 (15.7)	0.87	0.352
	Female	98 (61.6)	65 (40.9)	33 (20.7)		
Age (year)		56.14 ± 11.35	56.53 ± 10.29	55.45 ± 13.06	0.54	0.021
Size (cm)		1.07 ± 0.85	1.23 ± 0.94	0.79 ± 0.58	3.58	0.002
Composition	Solid	146 (91.8)	89 (56.0)	57 (35.8)	5.12	0.077
	Predominantly solid	11 (6.9)	10 (6.3)	1 (0.6)		
	Predominantly cystic	2 (1.3)	2 (1.3)	0 (0.0)		
Echogenicity	Hypoechogenicity	119 (74.8)	62 (39.0)	57 (35.8)	26.63	<0.001
	Isoechogenicity	40 (25.2)	39 (24.6)	1 (0.6)		
Orientation	Parallel	105 (66.0)	82 (51.5)	23 (14.5)	28.34	<0.001
	Nonparallel	54 (34.0)	19 (12.0)	35(22.0)		
Margin	Circumscribed	75 (47.2)	64 (40.3)	11 (6.9)	29.99	<0.001
	Not circumscribed	84 (52.8)	37 (23.3)	47 (29.5)		
Shape	Oval	67 (42.1)	60 (37.7)	7 (4.4)	36.84	<0.001
	Round	34 (21.4)	19 (12.0)	15 (9.4)		
	Irregular	58 (36.5)	22 (13.8)	36 (22.7)		
Calcification	Presence	111 (69.8)	82 (51.6)	29 (18.2)	17.00	<0.001
	Absence	48 (30.2)	19 (12.0)	29 (18.2)		
Posterior shadow	Presence	131 (82.4)	89 (56.0)	42 (26.4)	6.26	0.012
	Absence	28 (17.6)	12 (7.5)	16 (10.1)		
S-detect	Benign	76 (47.8)	69 (43.4)	7 (4.4)	46.72	<0.001
	Malignant	83 (52.2)	32 (20.1)	51 (32.1)		
ECI	Benign	89 (56.0)	82 (51.6)	7 (4.4)	140.29	0.181
	Malignant	70 (44.0)	19 (11.9)	51 (32.1)		
Radiologist	Benign	81 (50.9)	80 (50.3)	1 (0.6)	88.51	<0.001
	Malignant	78 (49.1)	21 (13.2)	57 (35.9)		
Total		159 (100)	101 (63.5)	58 (36.5)		

Unit: Person (%), Mean ± SD.

**Table 3:** Evaluation of the diagnostics performed by S-detect, ECI, and a radiologist.

Variable	AUC (95% CI)	Sensitivity	Specificity	Youden Index	PPV	NPV	*p* Value
S-detect	0.78 (0.72–0.84)	87.93	68.32	0.562	61.4	90.8	<0.001
ECI	0.85 (0.79–0.90)	87.93	81.19	0.773	72.9	92.1	<0.001
Radiologist	0.89 (0.84–0.93)	98.28	79.21	0.775	73.1	98.8	<0.001

ECI, elasticity contrast index; AUC, area under curve; CI, confidence interval; PPV, positive predictive value; NPV, negative predictive value.

**Table 4:** Diagnostic agreement of S-detect, ECI, and radiologist based on FNAC (*n* = 159).

Variable	Kappa	SE	95% CI	*p* Value
S-detect	0.52	0.06	0.38~0.64	<0.001
ECI	0.66	0.06	0.54~0.78	<0.001
Radiologist	0.72	0.05	0.62~0.83	<0.001

SE, standard error; CI, confidence interval; ECI, elasticity contrast index.

## Data Availability

The data supporting the findings of this study are available on request from the corresponding author (S.-H.Y.).

## Abbreviations

FNAC	fine-needle aspiration cytology
PPV	positive predictive value
NPV	negative predictive value
CAD	computer-aided diagnosis
ECI	elasticity contrast index
DL-CAD	deep learning-based computer-aided diagnosis
K-TIRADS	Korean Thyroid Imaging Reporting and Data System
SD	standard deviation
ROC	receiver operating characteristic
AUC	area under the curve
CI	confidence interval
SE	standard error
